# Concentrations of nicotine, nitrosamines, and humectants in legal and illegal cigarettes in Mexico

**DOI:** 10.1186/s12954-018-0257-3

**Published:** 2018-10-03

**Authors:** Ariela Braverman-Bronstein, James F Thrasher, Luz Myriam Reynales-Shigematsu, Mauricio Hernández-Ávila, Tonatiuh Barrientos-Gutierrez

**Affiliations:** 1 Center for Population Health Research, National Institute of Public Health, Av. Universidad 655, Col. Santa María Ahuacatitlán, 62100 Cuernavaca, Morelos Mexico; 2 Arnold School of Public Health, University of South Carolina, 921 Assembly St, Columbia, SC 29208 USA; 3 National Institute of Public Health, Av. Universidad 655, Col. Santa María Ahuacatitlán, 62100 Cuernavaca, Mexico

**Keywords:** Nicotine or derivatives, Tobacco constituents, Legislation

## Abstract

**Background:**

Article 10 of the World Health Organization Framework Convention on Tobacco Control states the need for industry disclosure of tobacco contents and emissions. Currently, the profiles of key tobacco compounds in legal and illegal cigarettes are largely unknown. We aimed to analyze and compare concentrations of nicotine, nitrosamines, and humectants in legal and illegal cigarettes collected from a representative sample of smokers.

**Methods:**

Participants of the International Tobacco Control cohort provided a cigarette pack of the brand they smoked during the 2014 wave. Brands were classified as legal or illegal according to the Mexican legislation. Nicotine, nitrosamines, glycerol, propylene glycol, and pH were quantified in seven randomly selected packs of each brand. All analyses were done blinded to legality status. Average concentrations per brand and global averages for legal and illegal brands were calculated. Comparisons between legal and illegal brands were conducted using *t* tests.

**Results:**

Participants provided 76 different brands, from which 6.8% were illegal. Legal brands had higher nicotine (15.05 ± 1.89 mg/g vs 12.09 ± 2.69 mg/g; *p* < 0001), glycerol (12.98 ± 8.03 vs 2.93 ± 1.96 mg/g; *p* < 0.001), and *N*-nitrosanatabine (NAT) (1087.5 ± 127.0 vs 738.5 ± 338 ng/g; *p* = 0.006) concentrations compared to illegal brands. For all other compounds, legal and illegal brands had similar concentrations.

**Conclusion:**

Compared to illegal cigarettes, legal brands seem to have higher concentrations of nicotine, NAT, and glycerol. Efforts must be made to implement and enforce Article 10 of the Framework Convention on Tobacco Control to provide transparent information to consumers, regulators, and policy-makers; and to limit cigarette engineering from the tobacco industry.

**Electronic supplementary material:**

The online version of this article (10.1186/s12954-018-0257-3) contains supplementary material, which is available to authorized users.

## Background

Articles 9 and 10 of the World Health Organization Framework Convention on Tobacco Control (WHO-FCTC) state that all countries must take action to ensure industry disclosure of tobacco contents and emissions as well as their regulation. [1] The minimal standards for reporting depend on the capacity of each country to measure tobacco compounds and emissions [2]. In 2016, the WHO-FCTC progress report found that of all the countries involved 67% are disclosing information on contents and 62% on emissions, while 54% regulate contents, and 50% emissions [3]. In the vast majority of countries, reports on tobacco products contents are limited to compounds defined in 1971 by the US Federal Trade Commission: nicotine and tar [4].

Monitoring of tobacco contents in cigarettes has been infrequent [5]. Nicotine [5, 6] and tobacco-specific nitrosamines across countries and cigarette brands have been measured, finding important differences even within the same brand [7–9]. Studies across countries have focused on one international brand (largely Marlboro Red) or on a single local brand per country. All previous studies have been restricted to legal brands, and to date, no study has reported these components in illegal cigarettes. Illegal products can represent up to 10% of the market [10], and the variability of sources for illegal tobacco could imply larger health risks, given that no standards of production are imposed [11].

On this paper, we aimed to analyze and compare a group of tobacco compounds that have been listed by the WHO as potential targets for regulation under Article 10 of the WHO-FCTC including legal and illegal brands from Mexico [12], collected from a representative sample of smokers. We focused in nicotine, the main addictive component and the four nitrosamines found in tobacco (*N*-nitrosoanabasine (NAB), 4-(methylnistrosamino)-1-(3-pyridyl)-1-butanone (NNK), *N*-nitrosonornicotine (NNN), and *N*-nitrosoanatabine (NAT)), considered to be the most carcinogenic tobacco constituents [13]. We also measured pH, which has been shown to be manipulated by the addition of compounds such as ammonia to increase the uptake of nicotine, [14] and two humectants (glycerol and propylene glycol), substances that have been known to be added or modified by the industry to improve the smoking experience [15]. Given their lack of regulation, we expected all of these compounds to be higher in illegal brands.

## Methods

### Sample

The International Tobacco Control Policy Evaluation Project (ITC Project) involves ongoing data collection from population-based cohorts of smokers in more than 25 countries in order to evaluate the key policies of the WHO-FCTC. The Mexican administration of the ITC (ITC Mexico) started in 2006 and has so far collected seven waves of data from representative sample of smokers in the following cities: Mexico City, Guadalajara, Tijuana, Monterrey, Puebla, and Leon, with approximately 280 participants per city each wave, with a larger sample in Mexico City, which is significantly larger than the other cities. The ITC Project methods have been previously described elsewhere [16].

For this study, data were collected in 2014 from ITC participants in Mexico City, Guadalajara, and Monterrey, the three largest cities in Mexico. These cities were selected based on their variability of cigarette brands [17], participation rates (80–90%) and their population density. Participants were asked to provide a new pack of their current cigarette brand at the time of the survey. A sample of 600 cigarette packs was estimated to be enough to have a minimum of 17 packs of the most common cigarette brands in Mexico [18–20]. This sample size allowed us to detect a significant difference between common cigarette brands in nicotine concentration of 3 ng/g with an alpha of 0.05 and an 80% power. Based on data for smokers’ preferred brands in prior surveys, we expected that this sample size would provide 150 packs of less common cigarette brands. The packs were identified with the survey id number of the participant in order to match the pack information with the subject’s survey data.

### Variables

#### Legality status

By law, legal cigarette brands must be registered in the Federal Official Gazette of Mexico. To classify cigarette brands as legal, we first reviewed if brands were registered in the Federal Official Gazette of Mexico on 2014. In addition, Mexican law requires cigarette packs to have a set of 4 pictograms occupying 30% of the front, warning labels on the side, a health message occupying 100% of the back side of the pack changing through the year, and the legend “to be sold exclusively in Mexico” [21]. Some cigarette packs of registered brands could have been legally produced in another country but smuggled into Mexico; to identify these cases, we further scrutinized pictograms and warning labels, to make sure they complied with the current Mexican legislation, and reviewed COFEPRIS warnings on illegal cigarettes and the lists containing illegal brand names [22]. If a pack complied with the law, it was classified as legal; otherwise, they were classified as illegal.

#### Quantitation of compounds

We received 770 packs from the ITC participants. Seven packs of each brand were randomly selected and submitted to the analysis of nicotine and humectants; in some cases, (9 legal and 13 illegal brands) less than seven packs were available, in such cases, we only analyzed the available packs (Additional file 1). In the case of nitrosamines, seven packs of the 10 most common legal and illegal brands were tested. We then estimated average concentrations for each brand and estimated the global average for legal and illegal brands using the average concentrations for each brand. All analyses were done blinded to the brand and legality status of the pack, and all the compounds, except for nitrosamines, were analyzed the Analytic Tobacco Compound Laboratory at The National Institute of Public Health of Mexico.

##### Nitrosamines

Nitrosamines (TSNAs) quantification was done by a private laboratory in NC, USA [23]. TSNAs were extracted from tobacco using an aqueous solution of ammonium acetate and shaken mechanically. Tobacco extracts were subsequently filtered and analyzed by liquid chromatography-mass spectrometry. The level of each TSNA present in a tobacco sample was quantified using one of two internal standards (d4-NNK and d4-NNN). D4-NNN was used to correct NNN, and d4-NNK was used to correct the other three TSNAs.

##### Nicotine

The process for nicotine quantitation followed the methods described by Coresta [24]. Briefly, after conditioning tobacco, nicotine was extracted with a mixture of n-hexane, sodium hydroxide solution, and water. The organic layer was analyzed by gas chromatography with a FID detector. The detection limit was 0.08 mg/g, and the quantitation limit was 0.25 mg/g.

##### Humectants

Glycerol and propylene glycol quantitation was performed according to AOAC International procedures [25]. Briefly, humectants were extracted from tobacco with a methanol solution. The extract was then analyzed with a spectrometer detector. The detection limits for glycerol and propylene glycol were 0.11 and 1.14 mg/g, respectively, and the quantitation limits were 0.17 and 1.57 mg/g respectively.

##### pH

For pH quantitation, deionized water was added to tobacco, after shaking it and letting it rest in the dark for 45 min, we decanted the supernatant and measured pH using a pH electrode with and internal reference of Ag/AgCl, previously calibrated with buffers of 4, 7, and 10 pH levels.

### Statistical analysis

We provide prevalence and 95% confidence intervals for the most commonly smoked brands considering the complex survey design. Descriptive statistics were calculated for each cigarette brand. Mean and standard deviations of each tobacco compound for legal and illegal brands were calculated. We computed *t* tests to compare the mean level of each compound between legal and illegal brands. A 0.05 significance level was defined for all tests. All analyses were performed using Stata 13.1 (College Station, TX, USA: StataCorp LP). R version 2.3.4 (The R Foundation for Statistical Computing Platform: x86_64-apple-darwin13.4.0 (64-bit)) was used to plot all graphs.

## Results

Participants provided packs for 76 different brands in total (Additional file 1). Most commonly smoked brands were legal (93%). Marlboro Red was the most common brand among those who smoke legal brands (29%), followed by Camel (14%), Montana Shots (13%), and Delicados (9%). As for the illegal brands, the most common one was Macpole (14%), followed by Blue River (10%), Rodeo (8%), and Sonora (7%) (Table 1). We did not detect smuggled packs of registered brands, such as Marlboro or Lucky Strike.

**Table 1:** Most common cigarette brand distribution and manufacturer by legal status (Mexico 2014)

Legal brands (manufacturer^a^) (93.1%)		Illegal brands^b^ (6.8%)	
Marlboro Red (PMI)	28.8 [22.6, 35.8]	Macpole	14.3 [4.7,36.3]
Camel (BAT)	13.9 [8.8, 21.2]	Blue River	9.9 [1.5,43.4]
Montana Shots (BAT	12.7 [8.9, 17.7]	Rodeo	7.9 [1.9,27.8]
Delicados (PMI)	9.5 [6.6, 13.4]	Sonora	6.8 [1.8,22.4]
Marlboro Gold (PMI)	6.3 [4.4, 8.9]	Laredo Menthol	6.1 [2.0,17.4]
Pall Mall Green Click-on (BTA)	5.1 [2.8, 9.4]	A One	5.3 [1.3,18.6]
Benson & Hedges Menthol (PMI)	4.2 [2.4, 7.1]	Marble Royal	4.1 [1.5,10.8]
Lucky Strike Red (BTA)	2.5 [1.2, 5.1]	GEM	3.7 [1.2,11.0]
Delicados Oval (PMI)	1.7 [0.7, 3.9]	Marble Gold	3.5 [0.8,13.6]
Benson & Hedges Gold (PMI)	1.6 [0.9, 2.8]	Capital	3.4 [0.7,14.7]
Marlboro Fresh (PMI)	1.5 [0.7, 3.1]	Seneca	3.3 [0.4,21.2]
Delicados Light (PMI)	1.4 [0.4, 4.3]	D&J	2.9 [0.7,11.1]
Marlboro Ice Xpress (PMI)	1.3 [0.5, 3.4]	Catalan	2.5 [0.3,16.6]
Garañon (GG)	1.3 [0.6, 2.8]	Laredo	2.3 [0.5,11.1]
Pall Mall White Click-on (BAT	1.2 [0.6, 2.4]	Link	2.3 [0.6,8.2]
Lucky Strike Additive Free (BAT)	1.0 [0.5, 2.0]	Ruby	2.1 [0.5,9.0]
Pall Mall Red (BAT)	0.9 [0.4, 2.0]	Black Jack	2.0 [0.5,6.8]
Pall Mall Ex White (BAT)	0.8 [0.3, 2.0]	Malverde	2.0 [0.5,7.8]
Pall Mall Fresh Click-on (BAT)	0.7 [0.3, 1.5]	Maypole Menthol	1.6 [0.4,6.5]
Pall Mall Red Click-on (BAT)	0.6 [0.1, 3.7]	Elite	1.4 [0.2,9.9]

% [95% CI]

^a^*PMI* Phillip Morris International, *BAT* British American Tobacco, *GG* Garañon Group

^b^There is no information on manufacturer for illegal brands

**Fig. 1:**
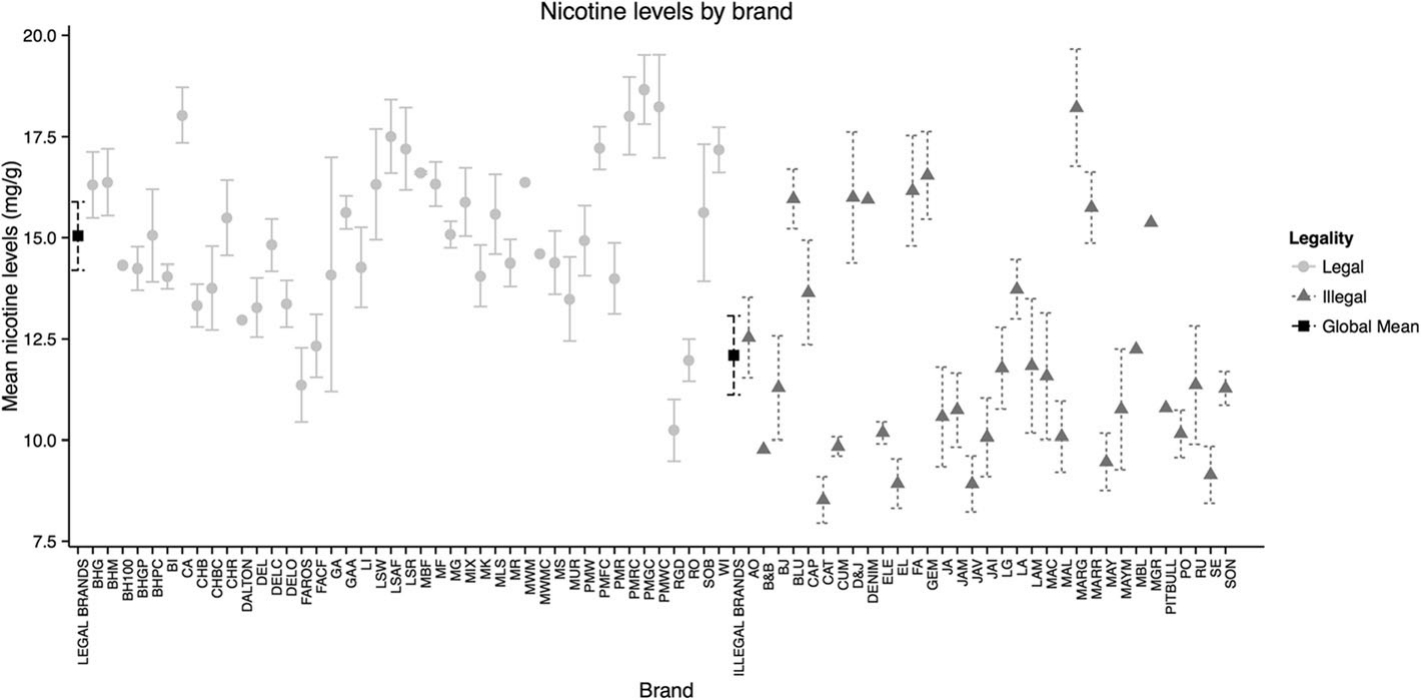
Mean nicotine levels by brand, according to legal status (Mexico 2014). The global mean of nicotine levels for legal brands was 15.5 mg/g and for illegal brands it was 12.1 mg/g

Figure 1 presents nicotine concentrations in legal and illegal brand varieties. Legal brands had higher nicotine concentrations (15.05 ± 1.90 mg/g) than illegal brands (12.10 ± 2.70 mg/g). The overall difference between legal and illegal brands was statistically significant (mean difference 2.95 mg/g; 95% CI (1.89, 3.99); *p* < 0.001).

**Fig. 2:**
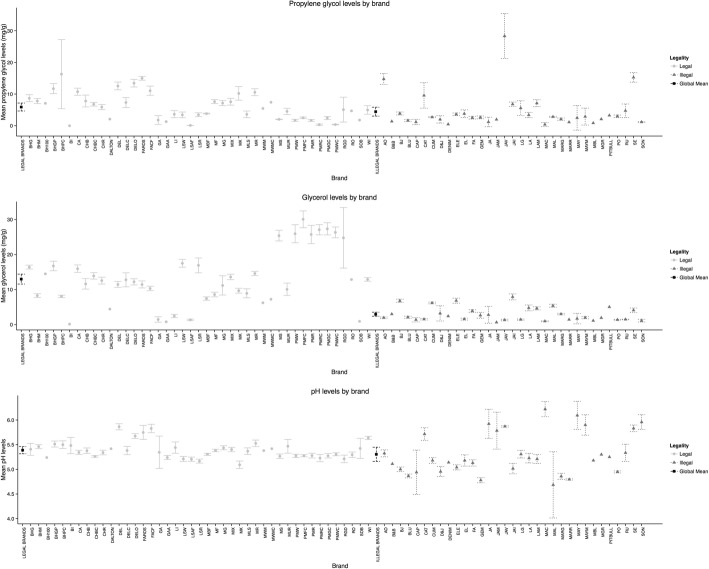
Humectants and pH mean levels by brand, according to legal status (Mexico 2014). The global mean of propylene glycol levels for legal brands were 5.9 mg/g and for illegal brands it was 4.44 mg/g. For glycerol, it was 12.98 mg/g for legal brands compared to 2.95 mg/g for illegal brands. And for pH, it was 5.39 for legal brands and 5.30 for illegal brands

Figure 2 shows the distribution of humectants and pH levels according to legal status. Legal and illegal brand varieties had similar propylene glycol levels, with a global mean for legal brands of 5.90 ± 4.23 mg/g and 4.44 ± 5.56 mg/g for illegal brands (*p* = 0.197). Legal brand varieties had higher glycerol levels, with a global mean of 12.98 ± 8.03 mg/g compared to 2.95 ± 1.96 mg/g for illegal brands (*p* < 0.001). Among legal brands, Pall Mall cigarettes had higher glycerol levels than other brand varieties. As for acid/base balance, pH levels were similar, for legal (5.39 ± 0.17) and for illegal (5.30 ± 0.42) brands (*p* = 0.239).

**Fig. 3:**
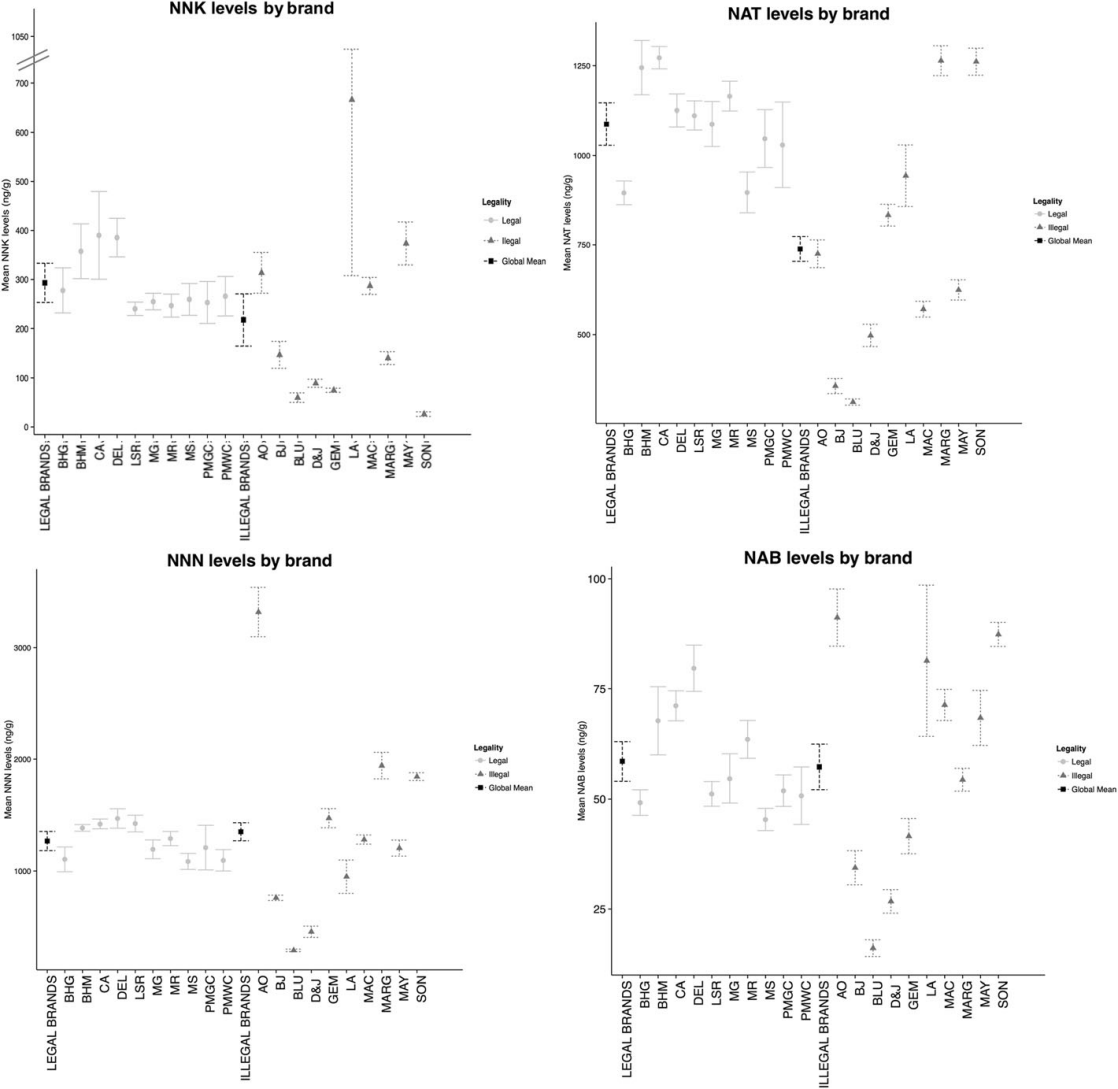
Nitrosamines mean levels by brand variety and legality (Mexico 2014). The global mean of NNK levels for legal brands was 293.1 mg/g, and for legal brands, it was 217.74 mg/g. For NAT levels, it was 1087.5 mg/g for legal brands, compared to 738.53 mg/g for illegal brands. For NNN levels, it was 1269.07 mg/g for legal brands and 1352.45 mg/g for illegal brands. And for NAB levels, it was 58.51 mg/g for legal brands and 57.28 mg/g for illegal brands

Figure 3 presents nitrosamine levels by brand and legal status. Legal and illegal brands were similar in their nitrosamine levels, with the exception of NAT, which in legal brands had a mean of 1087.50 ± 127.02 ng/g compared to that of 738.53 ± 338.14 ng/g in illegal brands (*p* = 0.007), the rest of the nitrosamines are summarized in Additional file 2.

## Discussion

We aimed to determine the concentrations of nicotine, pH, nitrosamines, and humectants in a representative sample of legal and illegal cigarette brands smoked in Mexico. We found that 6.8% of smokers living in the three largest metropolitan areas of Mexico provided illegal cigarette packs. Legal brands had higher concentrations of nicotine, glycerol, and NAT compared to illegal brands. Other nitrosamines, propylene glycol, and pH were similar between legal and illegal brands.

Nicotine is the primary addictive compound in cigarettes, while its health risks are not as high as other compounds, it is key to develop and sustain addiction [26]. We found that legal cigarette brands had a mean nicotine concentration of 15 mg/g, compared to 12 mg/g in illegal brands. Nicotine concentrations in Mexican cigarettes were studied previously in 2002 [5]. At that time, the dominant Marlboro Red cigarettes had 21 mg/g nicotine, and levels in the nationally produced brand Boots were lower (16 mg/g). In 1994 Benowitz, et al. [27] proposed gradually reducing nicotine levels over the course of 10–15 years to achieve a concentration of 0.17 mg of nicotine per cigarette to avoid reaching a daily dose of more than 5 mg of nicotine. Experimental studies show that slowly decreasing nicotine content in cigarettes is accompanied by a slow decrease in nicotine levels by smokers and a progressive decline in nicotine intake [28–31]. Based on our analyses, legal cigarettes in Mexico currently have 9–11 mg/cigarette, while illegal brands have 7–9 mg/cigarette, so currently, cigarette brands are 2 and 1.7 times above the recommended daily limit. Despite recent studies supporting Benowitz’s initial proposal [32], challenges for low-nicotine cigarettes prevail, mainly the opposition of tobacco companies [33]. The systematic analysis of nicotine in cigarettes provides transparency as to the addictive potential of cigarettes, making it urgent for Article 10 of the WHO-FCTC to be implemented.

Nitrosamines are one of the main carcinogenic compounds of tobacco [9]. We found that NAT concentrations were higher for legal compared to illegal brands, although levels for other nitrosamines were similar between legal and illegal cigarettes. A previous study of NNN and NNK levels in Mexican cigarettes found that the Marlboro Red brand cigarettes had a mean of 1.0 ìg/g of NNK + NNN levels [5]. We found that among legal brands, the levels of NNK + NNN were 1.5 ì g/g, while the concentration was 1.6g/g for illegal brands. Because nitrosamines are formed during tobacco processing, regulation of tobacco processing would likely reduce the amount of nitrosamines found in cigarettes. Several calls have been made to modify production standards to reduce nitrosamine contents. While this proposal is controversial [34], we consider that nitrosamines should be regularly measured as part of the regulatory tasks of health agencies across the world to better inform consumers about the health risks of smoking.

Additives such as humectants play an important role in smoking initiation and addiction by making the smoking experience more pleasant one [35]. We found that while propylene glycol levels were similar, legal brands had a glycerol concentration of 13 mg/g compared to that of 3 mg/g in illegal brands. Glycerol is used to increase shelf life and to improve the palatability of cigarettes [36, 37]. Cigarettes with additives are usually more appealing to smokers, since they enhance flavor and provide a smoother smoking experience; in addition, some additives mask the odor of *sidestream smoke* [38]. The harmful effects of humectants and added flavors are yet to be described. Recent evidence from e-cigarette research shows that flavors may affect nicotine absorption by modifying pH; in addition, they increase nicotine exposure though flavor liking [39, 40]. The regulation of flavors and humectants is also recommended by Article 9 of the WHO-FCTC^1^, and it might have an additional impact on the appeal and palatability of cigarettes for youth, as well as for promoting cigarette addiction.

Given their lack of regulation, we expected to observe higher concentrations of nicotine, nitrosamines, and humectants in illegal cigarettes. However, our results suggest that legal brands have higher concentrations of nicotine, NAT and glycerol, and comparable concentrations to illegal brands for all other compounds. We could not find previous references to explain this pattern, although we hypothesize that it could be linked to the role that legal and illegal cigarettes play in the market. Previous research has shown that illegal cigarette purchases tend to be unplanned and depend on availability and price [41, 42], *and could respond to tax avoidance* [43]. In contrast, legal brands have been known to engineer their products, including chemical composition, to ensure they attract and sustain their market share through standardized product characteristics, such as flavor and smoking experience.^38^ Nicotine and humectants have been referred to in legacy documents of the tobacco industry as components that could produce competitive advantage [37, 44]. Therefore, observed differences in these compounds could respond to engineering efforts aimed at sustaining the market share of legal brands in Mexico. An alternative explanation of differences could be confounding by type of cigarette. Regular and flavored cigarettes were found in both legal and illegal brands, but we only found light cigarettes in legal brands (Additional file 3). Since compound concentrations could differ by type of cigarette, we restricted compound comparisons between legal and illegal brands to regular cigarettes (75% of all packs). Within regular cigarettes, legal brands still had higher nicotine, glycerol, and NAT than illegal brands (Additional file 4).

Our study has some limitations worth mentioning. Our analyses were restricted to raw tobacco, with no evaluation of emissions, which limits the comparability of our results to an important proportion of the literature. Still, measuring compounds in raw tobacco is frequent, and studies measuring nicotine and nitrosamine using similar techniques have found comparable concentration levels [5, 45]. Also, current regulatory efforts are focusing in the concentration of nicotine in tobacco filler rather than in emissions, making our measurements relevant within a regulatory context [46, 47]. The eight components measured are far from a complete list of relevant constituents, and as such, these results should not be interpreted as a proof for illegal cigarettes to be safer, healthier, or better than legal ones; many other compounds could be present in higher concentrations in illegal cigarettes, including pesticides and metals [48, 49]. Indeed, prior research found that Mexican cigarettes had higher levels of arsenic than in other countries [50]. Still, we believe that measuring nicotine, nitrosamines, and humectants is an informative first step towards the implementation of Articles 9 and 10 of the WHO-FCTC. Lastly, by design, our sample of cigarette packs was obtained from a representative sample of smokers, and as such, it does not provide a complete list of all brands of cigarettes available in Mexico; our aim was to capture the characteristics of brands that are frequently used in the country, rather than producing a comprehensive characterization of all brands. Such task may be needed once regulatory agencies start evaluating compliance with product regulation, but it is beyond the scope of our paper.

Our results highlight the importance of developing better regulatory efforts and to implement controls for both legal and illegal tobacco. We found that nicotine, nitrosamines, and humectant concentrations are highly variable across legal brands, and in average appear to be higher than those observed in illegal cigarettes. Regulatory standards proposed in Article 9 of the FCTC to reduce the attractiveness, addictiveness, and toxicity of cigarettes through compound regulation could change this landscape if legal cigarettes are held to a maximum level of key compounds. Illegal cigarettes, however, will circumvent these regulations, requiring regulatory efforts to reduce the black market for cigarettes. Nicotine content regulation is currently being discussed by the FDA, in an effort to decrease cigarette addictiveness [47]. In our study, the lack of a regulatory framework allowed legal brands to have twice the nicotine concentration needed for addiction. Steps towards tobacco regulation and disclosure have been taken by the EU and the USA [46], and the WHO is developing guidelines to implement Articles 9 and 10 [2]. Establishing maximum permissible concentrations of key compounds and maintaining a strong, periodical, and independent monitoring system to assess compliance and provide public disclosure of tobacco contents and emissions will be needed to effectively implement Articles 9 and 10 of the FCTC.

## Conclusion

More than 48 billion cigarettes are sold in Mexico every year, yet, they are largely unregulated in terms of their contents and little is known about specific constituents [51]. In agreement with the WHO-FCTC, current legislation in Mexico enforces public smoking bans, requires warning labels in all cigarette packs, and bans all promotional advertising of cigarettes [19]. The General Law for Tobacco Control requires from tobacco companies to provide reports of tobacco constituents, but no independent assessment of the accuracy of those reports are conducted. As we move towards implementation of Article 10, it will be important to increase the capabilities of countries to conduct independent assessments of tobacco compounds and to establish funding strategies to maintain a surveillance system of tobacco constituents and emissions. Independent assessment will be key to eliminate interference from the industry.

## Additional files

Additional file 1: Brand varieties names, companies, abbreviations, and number of packs analyzed. Table describing the name of the brands, the abbreviation used in the manuscript, the company that produces such brand and the name of packs analyzed in the study. (DOCX 106 kb)

Additional file 2: Global mean for tobacco constituents according to legality status. Table describing the data reflected on the figures. Mean and standard deviation for each constituent among legal and illegal brands, and *t* test results to compare the groups. (DOCX 53 kb)

Additional file 3: Classification of brands according to type and legality status. Table describing the classification of the brands according to type of cigarettes: flavored, light, and regular. (DOCX 34 kb)

Additional file 4: Global mean for tobacco constituents for regular cigarette brands according to legality status. Mean and standard deviation for each constituent among legal and illegal brands, and *t* test results to compare the groups restricted only to regular brands. (DOCX 53 kb)
