# Supplementary material for: Concentrations of nicotine, nitrosamines, and humectants in legal and illegal cigarettes in Mexico
Source: Harm Reduct J. 2018 Oct 3;15:50. doi: 10.1186/s12954-018-0257-3 (PMC6171311; doi:10.1186/s12954-018-0257-3)
Supplement: Supplementary file 1 — Brand varieties names, companies, abbreviations, and number of packs analyzed. Table describing the name of the brands, the abbreviation used in the manuscript, the company that produces such brand and the name of packs analyzed in the study. (DOCX 106 kb) [file 12954_2018_257_MOESM1_ESM.docx]

Additional file 1: Brand varieties names, companies, abbreviations, and number of packs analyzed.

| **Legal** | | | | **Illegal** | | |
| --- | --- | --- | --- | --- | --- | --- |
| **Company** | **Brand variety** | **Abbreviation** | **# of packs** | **Brand variety** | **Abbreviation** | **# of packs** |
| Philip Morris International | Benson & hedges gold* | BHG | 7 | A one* | AO | 7 |
| Philip Morris International | Benson & hedges menthol* | BHM | 7 | Bulls & bears | B&B | 1 |
| Philip Morris International | Benson & hedges 100s | BH100 | 1 | Black jack* | BJ | 7 |
| Philip Morris International | Benson & hedges gold pearl | BHGP | 7 | Blue river* | BLU | 7 |
| Philip Morris International | Benson & hedges pearl capsule | BHPC | 3 | Capital | CAP | 3 |
| British American Tobacco | Bill | BI | 1 | Catalan | CAT | 7 |
| British American Tobacco | Camel* | CA | 7 | Cumbia | CUM | 4 |
| Philip Morris International | Chesterfield blue | CHB | 7 | D&J* | D&J | 7 |
| Philip Morris International | Chesterfield blue caps | CHBC | 7 | Denim | DENIM | 1 |
| Philip Morris International | Chesterfield red | CHR | 7 | El elegante | ELE | 7 |
| Dalton Corporation | Dalton | DALTON | 1 | Elite | EL | 7 |
| Philip Morris International | Delicados* | DEL | 7 | Farstar | FA | 7 |
| Philip Morris International | Delicados light | DELC | 7 | Gem* | GEM | 7 |
| Philip Morris International | Delicados oval | DELO | 7 | Jaipur | JA | 7 |
| Philip Morris International | Faros | FAROS | 7 | Jaipur menthol | JAM | 1 |
| Philip Morris International | FAROS with filter | FACF | 7 | Jaipur vanilla | JAV | 2 |
| Garañon Group | Garañon | GA | 7 | Jaisalmer menthol | JAI | 7 |
| Garañon Group | Garañon blue | GAA | 1 | L g premium | LG | 7 |
| British American Tobacco | Lucky strike white | LSW | 7 | Laredo* | LA | 7 |
| British American Tobacco | Lucky strike additive free | LSAF | 7 | Laredo menthol | LAM | 7 |
| British American Tobacco | Lucky strike red* | LSR | 7 | Link | LI | 7 |
| Philip Morris International | Marlboro black freeze | MBF | 2 | Macpole* | MAC | 7 |
| Philip Morris International | Marlboro fresh | MF | 7 | Malverde | MAL | 2 |
| Philip Morris International | Marlboro gold* | MG | 7 | Marble gold* | MARG | 7 |
| Philip Morris International | Marlboro ice xpress | MIX | 7 | Marble royal | MARR | 1 |
| Philip Morris International | Marlboro kretek mint | MK | 7 | Maypole* | MAY | 7 |
| Philip Morris International | Marlboro less smell | MLS | 7 | Maypole menthol | MAYM | 7 |
| Philip Morris International | Marlboro red* | MR | 7 | MONTERO blue | MBL | 1 |
| Philip Morris International | Marlboro white mint | MWM | 1 | MONTERO green | MGR | 1 |
| Philip Morris International | Marlboro white mint cápsula | MWMC | 1 | Pitbull | PITBULL | 1 |
| British American Tobacco | Montana shots* | MS | 7 | Police | PO | 4 |
| Tabaqueira | Muratti | MUR | 7 | Rodeo | RO | 1 |
| British American Tobacco | Pall mall ex white | PMW | 7 | Ruby | RU | 7 |
| British American Tobacco | Pall Mall Fresh Click on | PMFC | 7 | Seneca | SE | 7 |
| British American Tobacco | Pall mall red | PMR | 7 | Sonora* | SON | 7 |
| British American Tobacco | Pall Mall red Click on | PMRC | 7 |  |  |  |
| British American Tobacco | Pall Mall green Click on* | PMGC | 7 |  |  |  |
| British American Tobacco | Pall mall xl white click on* | PMWC | 7 |  |  |  |
| Ciroomex | Rgd | RGD | 7 |  |  |  |
| Garañon Group | Soberano | SOB | 1 |  |  |  |
| R.J Reynolds Tobacco Company | Winston | WI | 7 |  |  |  |

*Brands analyzed for nitrosamine levels.
