# Supplementary material for: Concentrations of nicotine, nitrosamines, and humectants in legal and illegal cigarettes in Mexico
Source: Harm Reduct J. 2018 Oct 3;15:50. doi: 10.1186/s12954-018-0257-3 (PMC6171311; doi:10.1186/s12954-018-0257-3)
Supplement: Supplementary file 2 — Global mean for tobacco constituents according to legality status. Table describing the data reflected on the figures. Mean and standard deviation for each constituent among legal and illegal brands, and t test results to compare the groups. (DOCX 53 kb) [file 12954_2018_257_MOESM2_ESM.docx]

Additional file 2. Global mean for tobacco constituents according to legality status.

|  | **Legal** | **Illegal** | **Mean Difference** | **P value^a^** |
| --- | --- | --- | --- | --- |
|  | **Mean (sd)** | **Mean (sd)** |  |  |
| Propylene glycol | 5.90 (4.23) | 4.44 (5.56) | 1.46 | 0.197 |
| pH | 5.39 (0.17) | 5.30 (0.42) | 0.09 | 0.239 |
| Nicotine | 15.05 (1.90) | 12.10 (2.70) | 2.95 | <0.001 |
| Glicerol | 12.98 (8.03) | 2.95 (1.96) | 10.03 | <0.001 |
| NAB | 58.51 (11.29) | 57.28 (26.58) | 1.23 | 0.894 |
| NNK | 293.10 (59.79) | 217.74 (196.86) | 75.36 | 0.262 |
| NAT | 1087.50 (127.02) | 738.53 (338.14) | 348.97 | 0.007 |
| NNN | 1269.07 (150.13) | 1352.45 (879.15) | -83.38 | 0.771 |

a = T-test.
