# Supplementary material for: Concentrations of nicotine, nitrosamines, and humectants in legal and illegal cigarettes in Mexico
Source: Harm Reduct J. 2018 Oct 3;15:50. doi: 10.1186/s12954-018-0257-3 (PMC6171311; doi:10.1186/s12954-018-0257-3)
Supplement: Supplementary file 3 — Classification of brands according to type and legality status. Table describing the classification of the brands according to type of cigarettes: flavored, light, and regular. (DOCX 34 kb) [file 12954_2018_257_MOESM3_ESM.docx]

Additional file 3: Classification of brands according to type and legality status

|  | **Legal*** | **Illegal** |
| --- | --- | --- |
|  | **43 (%)** | **33 (%)** |
| **Regular** | 22 (51.2) | 28 (84.8) |
| **Flavored** | 15 (34.9) | 5 (15.2) |
| **Light** | 1. (20.9) | 0 |

*Some brands were both flavored and light
