# Supplementary material for: Concentrations of nicotine, nitrosamines, and humectants in legal and illegal cigarettes in Mexico
Source: Harm Reduct J. 2018 Oct 3;15:50. doi: 10.1186/s12954-018-0257-3 (PMC6171311; doi:10.1186/s12954-018-0257-3)
Supplement: Supplementary file 4 — Global mean for tobacco constituents for regular cigarette brands according to legality status. Mean and standard deviation for each constituent among legal and illegal brands, and t test results to compare the groups restricted only to regular brands. (DOCX 53 kb) [file 12954_2018_257_MOESM4_ESM.docx]

Additional file 4. Global mean for tobacco constituents for regular cigarette brands according to legality status.

|  | **Legal** | **Illegal** | **Mean difference** | **p value^a^** |
| --- | --- | --- | --- | --- |
|  | **n=22** | **n=28** |  |  |
|  | **mean (sd)** | **mean (sd)** |  |  |
| pH | 5.43 (0.20) | 5.26 (0.42) | 0.17 | 0.071 |
| Propylene Glycol | 5.88 (4.56) | 3.54 (3.72) | 2.34 | 0.052 |
| Glycerol | 10.45 (7.37) | 2.89 (1.79) | 7.56 | <0.001 |
| Nicotine | 14.59 (1.98) | 12.38 (1.98) | 2.21 | 0.003 |
| NAB | 60.00 (13.63) | 57.27 (26.58) | 2.73 | 0.820 |
| NNK | 299.90 (69.21) | 217.74 (196.86) | 82.16 | 0.346 |
| NAT | 1077.76 (151.58) | 738.52 (338.14) | 339.24 | 0.037 |
| NNN | 1300.48 (169.76) | 1352.45 (879.15) | -51.97 | 0.889 |

a: T-test
